# Group A streptococcal (GAS) infections amongst children in Europe: Taming the rising tide

**DOI:** 10.1016/j.nmni.2022.101071

**Published:** 2022-12-15

**Authors:** Nityanand Jain, Edouard Lansiaux, Aigars Reinis

**Affiliations:** Faculty of Medicine, Riga Stradinš University, 16 Dzirciema street, Riga, LV-1007, Latvia; Joint Laboratory, Pauls Stradinš Clinical University Hospital, 13 Pilsonu street, Riga, LV-1002, Latvia; Lille University School of Medicine, 2 Avenue Eugène Avinée, 59120 Loos, Lille, France; Faculty of Medicine, Riga Stradinš University, 16 Dzirciema street, Riga, LV-1007, Latvia; Joint Laboratory, Pauls Stradinš Clinical University Hospital, 13 Pilsonu street, Riga, LV-1002, Latvia

**Keywords:** Death, France, Guidelines, Infections, Management, UK

## Abbreviations

GAS –: Group A streptococcal infections
iGAS –: invasive Group A streptococcal infections
CFR –: case fatality rate
UKHSA –: United Kingdom Health Security Agency
WHO –: World Health Organization
ECDC –: European Centre for Disease Prevention and Control

Editorial

**Fig. 1:**
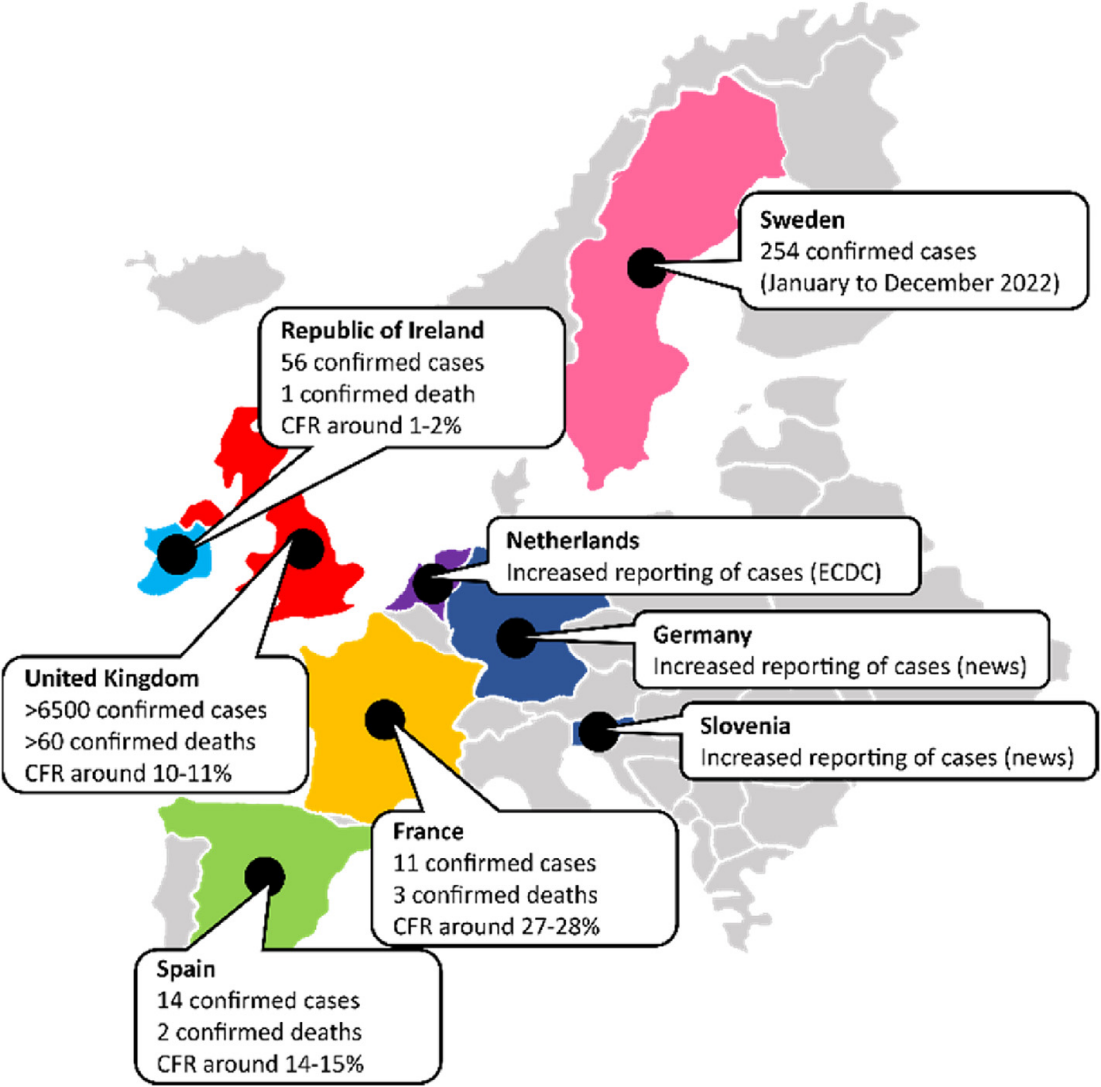
Number of confirmed cases and deaths of Group A Streptococcal infections (both for Scarlet fever and invasive forms combined). CFR: case fatality rate. According to ECDC/WHO (European Centre for Disease Prevention and Control/World Health Organization), Ireland, UK, France, Netherlands, and Sweden are the countries experiencing increased GAS notifications. Spain was removed from this list by ECDC based on a comparison with previous years. GAS notifications are reported to be higher in Germany and Slovenia based on statements from local epidemiologists in newspapers. Note that the authors remain neutral in regards with territorial depictions used in the map. Data source: Ministry of Health of respective countries.

Since the beginning of December 2022, unusually high numbers of cases and deaths among children have been reported across the United Kingdom (UK) and other regional European countries like Ireland, France, Netherlands, Spain, and Sweden (Fig. 1) [1,2]. The sudden increase seems to be caused by the rapid spread of Group A Streptococcus (GAS) infections. Generally, it is common to see an uptick in the number of case notifications of GAS infections during this time of the year (in some countries). However, this year the numbers being reported are much higher when compared week-to-week with previous years. It is pertinent to note that the number of weekly cases has not yet reached the peak levels from the past years [3].

**Fig. 2:**
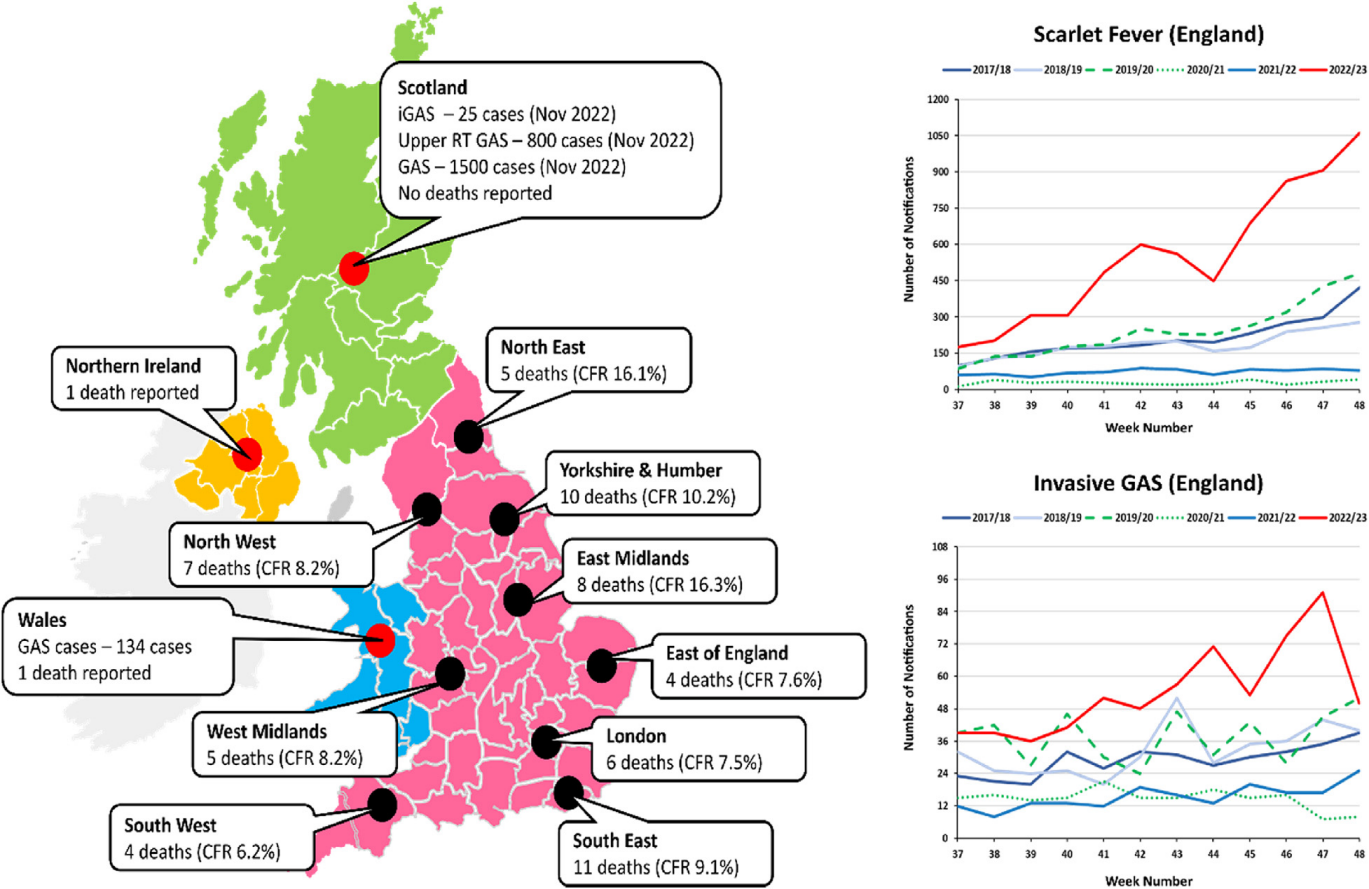
Map (left) showing the case fatality rate (CFR) and number of deaths in different regions of United Kingdom (UK) as of 7th December 2022. Upper RT stands for upper respiratory tract. Note that the authors remain neutral in regards with territorial depictions used in the map. Graphs (right) showing the total number of notifications in England for Scarlet fever and invasive group A Streptococcal infection (iGAS) from week 37 to week 48 (mid-September to early-December). A season runs from week 37 to week 36 each year (mid-September to mid-September). Data Source: UKHSA (https://www.gov.uk/government/publications/group-a-streptococcal-infections-activity-during-the-2022-to-2023-season/group-a-streptococcal-infections-first-update-on-seasonal-activity-in-england-2022-to-2023).

The United Kingdom has been the worst affected country by this sudden rise. As of 7th December 2022, England alone has reported over 6,600 cases of scarlet fever (an illness that mainly affects children caused by GAS infections) in a period of just 12 weeks (averaging around 550 new cases per week) along with another 652 cases of invasive GAS infections. About 60 cases of deaths have been confirmed in the same period with a mean case fatality rate (CFR) of 9.92% across the country. Age-based stratification has revealed the highest CFR amongst 10-14 years old (27.3%), followed by elderly aged ≥75 years (16.3%). Scotland, on the other hand, has reported cases that were like those observed at the peaks in previous pre-COVID-19 pandemic years (Fig. 2).

### 1 Group A streptococcus (GAS) infections

**Fig. 3:**
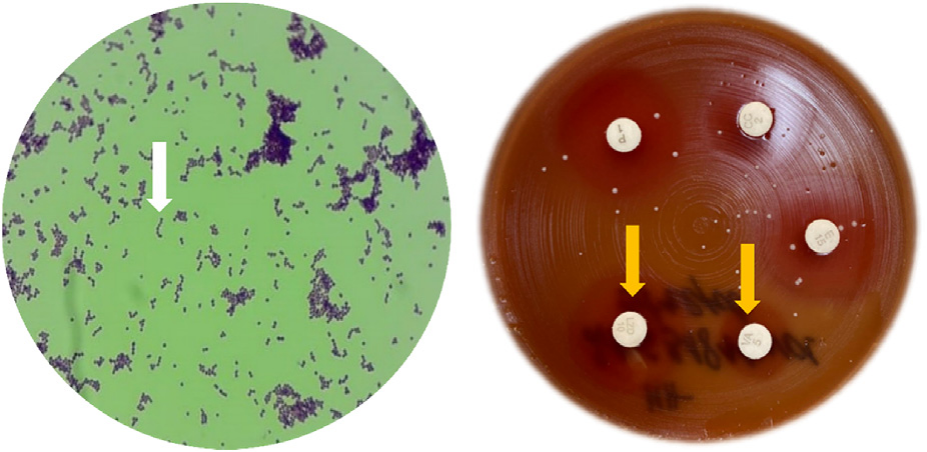
Gram-staining microscopy (left) for *S. pyogenes* sample isolated from blood sample. The microbe forms chains (white arrow) and is Gram positive. β-haemolytic changes (right; yellow arrows) seen on blood agar on cultivation of *S. pyogenes* colonies. Note the presence of minor contamination on the blood agar plate from an undetermined source.

*Streptococcus pyogenes*, commonly referred to as Group A beta-haemolytic Streptococcus, is a Gram-positive, non-motile, resident bacteria of the normal human skin, nasopharyngeal, and anogenital tract microflora. Under the microscope, *S. pyogenes* is visualized as occurring in pairs or chains of multiple round-to-ovoid cocci, each approximately 0.6-1.0 μm in diameter [4]. The bacteria often needs a complex enriched medium like blood agar to grow, where beta-haemolytic changes (clear and complete lysis of red blood cells surrounding the colony) can be seen (Fig. 3). As an exclusively human pathogen, *S. pyogenes* has been known to colonize the pharynx of asymptomatic individuals. The carriage rate is typically highest amongst asymptomatic school-aged children (5-15 years), ranging from 8.4-12.9% in high-income countries to 15-20% in developing countries [5].

The bacteria has been traditionally thought to spread by large respiratory droplets (when coughing, sneezing, or talking) from infected individuals, including asymptomatic carriers [6]. However, with advances in the methodological approaches, additional modes of transmission have been uncovered. Nasal secretions, sputum or spit, dust particles, skin-to-skin direct contact, indirect contact with surfaces or beddings/fabrics, food, and biological vectors like insects have now been proven to promote the transmission of bacteria [6].

Clinically, it can cause a myriad of symptoms like tonsillitis, pharyngitis, scarlet fever, impetigo, erysipelas, cellulitis, and pneumonia [7]. The bacteria can cause both invasive and non-invasive forms of disease. In 2005, using conservative estimation methodologies, WHO reported that globally more than 18 million individuals are affected with GAS infections, with an annual increase of more than 1.7 million new notified cases and 500,000 deaths [8]. This makes GAS infections the ninth leading cause of human mortality.

The risk factors for GAS infections include inadequate housing, homelessness, overcrowding, sharing of personal items, poor ventilation, and low socioeconomic status. In hospital settings, poor infection control practices, cross-contamination of devices and equipment, poor personal hand hygiene, and exposure to asymptomatic individuals are considered significant risk factors. Changes in immune status due to pregnancy, chronic diseases, malnutrition, and substance use have also been highlighted as risk factors [9]. Boys tend to be more at risk than girls. The most common environments for transmission include schools, kindergartens, hospitals, military training facilities, and care homes [9].

Superficial GAS infection manifestations include pharyngitis, scarlet fever (scarlatina), and impetigo (Table 1) [10]. For pharyngitis, the UKHSA recommends to use FeverPAIN and CENTOR criteria to determine whether to prescribe antibiotics or not (FeverPAIN score of 3 or more is an indication for antibiotic prescription). Additionally, GAS infections can trigger post-infection autoimmune reactions causing acute post-streptococcal glomerulonephritis, acute rheumatic fever, and/or rheumatic heart disease [10]. Bacteraemia and cellulitis are the most common forms of invasive GAS (iGAS) infections. Mortality in iGAS infections remains high with 8-23% of individuals dying within a week of infection [10]. Rarely iGAS can manifest as necrotizing fasciitis, septic arthritis, pneumonia, meningitis, abscess, osteomyelitis and other focal infections, endocarditis, and peritonitis [10].

**Table 1:** Clinical manifestations of superficial GAS infections

Disease	Clinical Manifestations^a^
Pharyngitis	• sudden-onset fever (>38.3° C) accompanying a sore throat • inflammation of the pharynx and tonsils • patchy exudates and cervical lymph node adenopathy • other symptoms - malaise, fever, headache, nausea, abdominal pain, and vomiting
Scarlet Fever	• accompanied by exudative GAS pharyngitis (first to appear) • 12-48 hours later develops deep red, finely papular, erythematous rash (sandpaper-like feeling) • Rash appears first on chest and stomach, rapidly spreading to other body parts • strawberry tongue • peeling skin on the fingertips, toes, and groin area, as the rash fades
Impetigo	• pustules that gradually enlarge and rupture, forming thick, honey-coloured scabs

a Patient can be evaluated using the FeverPAIN score (available at https://www.mdcalc.com/calc/3316/feverpain-score-strep-pharyngitis) or Centor score (available at https://www.mdcalc.com/calc/104/centor-score-modified-mcisaac-strep-pharyngitis).

### 2 Clinical management & prophylaxis

Management of *S. pyogenes* requires the administration of antibiotic therapy (oral formulations are preferred – tablets/capsules), with penicillins being the first choice [7]. Global resistance to penicillins remains low, though some cases of rare mutant strains that are resistant to penicillins have been reported [11]. Alternatively, in cases of penicillin allergies or intolerance, macrolides and clindamycin have been the first choice. However, antimicrobial resistance against both these drugs presents a challenge [12].

According to the interim guidelines from the UK Health Security Agency (UKHSA), co-trimoxazole (SXT; antifolate therapy) is recommended as the second line in case of penicillin allergy (Table 2). However, recent research has shown that when blocked by SXT from producing its own folates, *S. pyogenes* (isolated from clinical samples) is capable of overcoming susceptibility to SXT by uptaking reduced forms of folic acid directly from the host cells [13]. This makes the antibiotic ineffective and the patient will likely deteriorate despite antibacterial therapy.

**Table 2:** Summary of interim clinical guidelines released by UKHSA (UK Health Security Agency) on 09th December 2022 (valid till end of January 2023)

Key points from UKHSA interim clinical guidance (09.12.2022 – 31.01.2023)	
**Management of Possible Group A Streptococcus (GAS)**	• Oral Phenoxymethylpenicillin is the first line of choice. • Amoxicillin, Macrolides and Cefalexin (in order of decreasing preference) can be considered as alternatives in case of non-availability of penicillin. • In case of non-severe penicillin allergy, Macrolides and Cefalexin are alternatives. • In case of severe penicillin allergy, Macrolides and Co-trimoxazole (SXT) are alternatives. • A 5-day course is enough for symptomatic cure. However, a longer 10-day course is needed for microbiological cure. The final decision of duration lies with treating clinician.
**Management of Invasive Group A Streptococcus (iGAS)**	• Maintain a low threshold for considering pulmonary complications of GAS. • Prompt initiation of appropriate antibiotics remains key. • Take a throat swab, blood cultures and other appropriate samples including respiratory culture, tissue, and fluid samples. • For culture-negative fluid specimens, it is advised to use PCR (GAS specific or 16S rDNA) for confirmation.
**Notification of Cases (Epidemiology)**	• Both GAS and iGAS are notifiable (both suspected and confirmed cases). • Definition for Severe GAS: a) Invasive disease defined through the isolation of GAS from a normally sterile site; b) GAS is isolated from a non-sterile site in combination with clinical signs consistent with a severe infection (streptococcal toxic shock syndrome, pneumonia, necrotising fasciitis, puerperal sepsis, meningitis, septic arthritis). • Diagnosis can be made using both culture and molecular methods.
**Management of Contacts (Contact Tracing)**	• Defined as prolonged contact with the case in a household-type setting during the 7 days before onset of symptoms and up to 24 hours after initiation of appropriate antimicrobial therapy in the index case. • Following individuals are required to undergo antibiotic prophylaxis following close contact: a) Pregnant women from ≥37 weeks gestation; b) Neonates and women within the first 28 days of delivery; c) Older household contacts (≥75 years); d) Individuals who develop chickenpox with active lesions either seven days prior to onset in the iGAS case or within 48 hours after the iGAS case commences antibiotics if exposure is ongoing.
**Special Management in School or Early-year Settings**	• Health Protection Teams or HPTs should be immediately contacted when schools experience any one of the following situations: a) one or more cases of chickenpox or Influenza in the class that has scarlet fever at the same time; b) outbreak of scarlet fever in a setting/class that provides care or education to children who are clinically vulnerable; c) outbreak continues for over 2 weeks, despite taking steps to control it; d) any child or staff member is admitted to hospital with any GAS infection (or there is a death); e) any issues that are making it difficult to manage the outbreak. • Further guidelines for schools is available from https://www.gov.uk/government/publications/scarlet-fever-managing-outbreaks-in-schools-and-nurseries (Accessed 10th December 2022).

Additionally, the antibiotic shortage has been a growing concern in the affected countries. French National Agency for Medicines and Health Products Safety (ANSM) has notified that there is a significant national strain on the supply of Amoxicillin, especially the paediatric formulations and dosages. ANSM further recommends not to prescribe antibiotics in cases of unconfirmed diagnosis, or viral diseases [14]. Across the English Channel, in the UK, the chief pharmaceutical officer for NHS (National Health Service) England, described intermittent disruptions in the supply of liquid penicillin but confirmed that sufficient stocks were available at a national level.

However, the situation on the ground is far from being portrayed by the authorities. Local pharmacies have raised concerns regarding increased demands of β-lactams due to lower prescription thresholds and use in the prophylaxis of contacts (Table 2). Pharmacies are being forced to provide these antibiotics at a loss, even though experts have questioned the need for lowering prescription thresholds [15]. Since the rise in GAS notifications outmatched iGAS notifications, there is evidence that GAS infections are not rapidly progressing to invasive disease forms. Furthermore, such widespread use of penicillins risks the development of increased community-level antimicrobial resistance.

To alleviate the situation, in a joint communique, the Royal College of General Practitioners (RCGP), Royal College of Paediatrics and Child Health (RCPCH), and Royal College of Emergency Medicine (RCEM) have stressed that GAS infection is both common and treatable with most children presenting with mild symptoms or asymptomatic course of the disease. Majoritarily, children recover on their own without the need for empirical antibiotic therapy [16]. Furthermore, if treated, children are not infectious after 24-48 hours of the start of antibiotics and can return to school once they are feeling well enough after this period [17]. However, if left untreated, children can be contagious for 10-21 days.

RCGP's chair, Dr. Kamila Hawthorne, has also supported calls for allowing pharmacists to change prescriptions based on stocks and needs – “*Not only do pharmacists need to be able to dispense a different formulation of that antibiotic, but if that antibiotic is just not available there are other alternatives that are just as good”* [18]. On similar lines, ANSM has recommended a shorter 5-day course for treatment for symptomatic children.

### 3 Why the sudden rise this season?

The UKHSA has reported that there is currently no evidence of a new mutant strain circulating in the society. According to Professor Shiranee Sriskandan at the Imperial College London's Centre for Bacterial Resistance Biology (CBRB), since the past few years, there has been an annual surge in the number of GAS notifications in the UK due to the expansion and dominance of fitter strains than the previously dominant versions [19]. Of note is that the UKHSA reported that “*The current emm types have been circulating for the last five years, following the documented emergence of M1*_*UK*_ *in 2016*” (Box 1) [20,21].

Box 1 **Description of the M1UK strain.**

**Table:** 

What is the M1_UK_ strain?
GAS strains are classified based on the *emm* type that is determined based on the amino acid sequence of a protein found on the bacterial cell wall (M protein). There are currently >220 known strains based on *emm* classification. In developed countries, the most common *emm* types include *emm1*, *emm3*, *emm12*, and *emm28*. These are also the types mostly isolated from asymptomatic carriers. Based on disease severity, *emm1, emm3*, and *emm49* are associated with invasive disease whilst *emm2, emm4, emm6, emm12*, and *emm44/61* are associated with superficial disease. M1_UK_ (M1T1 *S. pyogenes*) is a mutant strain of GAS within the *emm1* family that was discovered in 2019 but has been in circulation since 2010. The strain differs by just 27 mutations when compared with other *emm1* family strains which allows it to increase the production of streptococcal pyrogenic exotoxin A (SpeA) by more than nine-times. This makes the strain fitter and more dominant in the society. It was designated as the largest single contributor to both non-invasive and invasive infections in 2016.

Alt-text: Box 1

Furthermore, COVID-19 related global lockdowns had significantly reduced social interactions and confinements in small spaces (classrooms), whilst accelerating the adoption of non-pharmaceutical interventions (NPIs) like hand hygiene, use of face mask and other. These positive measures helped in keeping the load of many viral and bacterial paediatric infections low during the pandemic [22,23]. However, on the other side, it led to reduced immune stimulation (due to reduced transmission and delayed contact with microbial agents) and lower vaccine uptake, thereby causing a so-called “immune debt” [23]. In fact, it has been argued that longer the periods of lockdowns, the greater the risk of future epidemics (rebound effect) due to growing proportion of susceptible individuals and declining herd immunity [23].

Now that the pandemic-era restrictions have been lifted, the increase in social mixing (increased nodes of transmission, thereby increasing microbial load in the environment) and confinement to indoor spaces due to winter can be a plausible explanation of this sudden rise. Additionally, since GAS notifications were lower during pandemic time, we now have a double cohort of non-immune children interacting with the bacteria, thereby causing infections [23]. This, nevertheless, is a matter of serious concern given the concomitant rise in notifications of other respiratory infections including influenza and RSV (respiratory syncytial virus) [22].

In conjunction with the immune-debt theory, another theory proposes that COVID-19 infections (in both symptomatic and asymptomatic cases) could have led to immune dysregulation in children (so called “immune theft”), thereby leaving children susceptible to subsequent infections. As for increased iGAS notifications, the UKHSA suspects it to be consequence of heightened scarlet fever activity. Genomic analysis has previously shown a crossover of strains associated in both presentations [24,25].

### 4 Taming the rising tide

A multisectoral and multiprong strategy is needed to curb the rising cases of GAS infections. Firstly, measures for primary prevention need to be introduced including epidemiological investigations and improved surveillance systems. Prompt identification and notification of clusters require a coordinated response from general practitioners (GPs), pharmacists, parents and schools since they are the first contact with children. This requires public education and outreach. A free-to-complete online module is available for GPs for training (Espresso CPD: invasive Group A streptococcus; available from https://www.mimslearning.co.uk/courses/espresso-cpd-invasive-group-a-streptococcus).

Personal protective measures should be continued and encouraged including good hand hygiene and avoiding overcrowding. Limiting sharing of personal items like water bottles, drinking glasses, beddings, toiletries etc., should be encouraged [9]. Disinfection of surfaces is equally crucial. Alcohol based disinfectants like 4% formaldehyde, 2% glutaraldehyde, 70% ethanol, 70% propanol and others., have been effective in GAS disinfection. GAS also seems susceptible to moist heat of 121 °C for at least 15 min and/or dry heat of 170 °C for at least 1 h [9,26]. Use of personal protective suits and respirators is essential in hospitals and other high-risk environments.

Screening of contacts and initiation of post-exposure prophylaxis should be done based on national guidelines. Screening should include swabs from both throat and perianal region [27]. Early initiation of antimicrobial therapy is crucial to prevent transmission and severe course of disease. Whole genome sequencing can enable the early detection and taming of GAS infections and the identification of outbreaks. For example, the US CDC's (centre for disease control and prevention) Active Bacterial Core surveillance (ABCs) uses genome sequencing to track new strains and antibiotic resistance trends.

Presently, there is no vaccine available for GAS prophylaxis though multiple candidates are under different phases of development. The main challenge stems from constantly emerging newer and more resistant strains (due to frequent genetic recombination events, including gene exchange and single nucleotide polymorphisms) [28]. Additionally, there is heightened risk of immune cross-reactivity with cardiac proteins. A detailed update on the status of different candidate vaccines is presented by Castro and Dorfmueller [28].

We would like to also draw attention towards the need to tame the misinformation campaigns that have somewhat become non-separable from any infectious endemic. Following the widespread rise in new cases and deaths, multiple false pieces of information are being circulated on various social media platforms. Amongst them, the most circulated piece is a study published in 2014 that reported on the possible increase in colonization of *Streptococcus pneumoniae* and *Staphylococcus aureus* in mice following immunization by live attenuated influenza vaccine [29]. The authors reported that following vaccination, there is a significant decrease in normal bacterial clearance from nasopharynx that leads to increased bacterial carriage densities and durations [29]. However, *S. pneumoniae* does not belong to GAS group of bacteria.

In conclusion, we would urge the public-at-large to follow official guidelines, be vigilant of early red-flag symptoms of GAS infections, do not fall for misinformation, get vaccinated against COVID-19 and influenza and undertake other precautionary measures as discussed above. Authorities have expressed hope that moving forward, as life returns to pre-pandemic standards, the immune debt would be paid off and cases would begin to drop.

### Ethical approval

Not applicable. All data presented in the paper has been collected from open-source platforms with proper citation and/or from media sources.

### Conflicts of interest

The authors declare no conflicts of interest in regards with the present paper.

### Funding

The present paper didn't receive any external funding and was self-supported by the authors.

### Author contributions

NJ and EL conceptualized the paper whilst all authors were involved in data collection, writing, correction, and finalizing of the final draft. Visualization was done by NJ whilst supervision was done by AR. All authors have read and agreed to the final version for publication.
